# Applications of Site-Specific Labeling to Study HAMLET, a Tumoricidal Complex of α-Lactalbumin and Oleic Acid

**DOI:** 10.1371/journal.pone.0026093

**Published:** 2011-10-10

**Authors:** Natalia Mercer, Boopathy Ramakrishnan, Elizabeth Boeggeman, Pradman K. Qasba

**Affiliations:** 1 Structural Glycobiology Section, CCR-Nanobiology Program, Center for Cancer Research, NCI-Frederick, Frederick, Maryland, United States of America; 2 Basic Science Program, SAIC-Frederick, Inc., NCI-Frederick, Frederick, Maryland, United States of America; Instituto de Biociencias - Universidade de São Paulo, Brazil

## Abstract

**Background:**

Alpha-lactalbumin (α-LA) is a calcium-bound mammary gland-specific protein that is found in milk. This protein is a modulator of β1,4-galactosyltransferase enzyme, changing its acceptor specificity from *N*-acetyl-glucosamine to glucose, to produce lactose, milk's main carbohydrate. When calcium is removed from α-LA, it adopts a molten globule form, and this form, interestingly, when complexed with oleic acid (OA) acquires tumoricidal activity. Such a complex made from human α-LA (hLA) is known as HAMLET (Human A-lactalbumin Made Lethal to Tumor cells), and its tumoricidal activity has been well established.

**Methodology/Principal Findings:**

In the present work, we have used site-specific labeling, a technique previously developed in our laboratory, to label HAMLET with biotin, or a fluoroprobe for confocal microscopy studies. In addition to full length hLA, the α-domain of hLA (αD-hLA) alone is also included in the present study. We have engineered these proteins with a 17–amino acid C-terminal extension (hLA-ext and αD-hLA-ext). A single Thr residue in this extension is glycosylated with 2-acetonyl-galactose (C2-keto-galactose) using polypeptide-α-*N*-acetylgalactosaminyltransferase II (ppGalNAc-T2) and further conjugated with aminooxy-derivatives of fluoroprobe or biotin molecules.

**Conclusions/Significance:**

We found that the molten globule form of hLA and αD-hLA proteins, with or without C-terminal extension, and with and without the conjugated fluoroprobe or biotin molecule, readily form a complex with OA and exhibits tumoricidal activity similar to HAMLET made with full-length hLA protein. The confocal microscopy studies with fluoroprobe-labeled samples show that these proteins are internalized into the cells and found even in the nucleus only when they are complexed with OA. The HAMLET conjugated with a single biotin molecule will be a useful tool to identify the cellular components that are involved with it in the tumoricidal activity.

## Introduction

Alpha-lactalbumin (α-LA) is a 14 kDa, Ca^2+^-binding milk protein, synthesized in the secretory cells of lactating mammary glands. Its main function is to interact with β1,4-galactosyltransferase-1 (β4Gal-T1) to form lactose synthase complex (LS). By binding to β4Gal-T1, α-LA changes the acceptor specificity of β4Gal-T1 from GlcNAc to glucose, to synthesize lactose, which is the primary carbohydrate in milk of most mammalian species [1]. Due to the similarities in gene structure and protein sequences, it has been proposed that α-LA and c-type lysozyme have evolved from the same gene [2]. As in the protein structure of c-type lysozyme, α-LA has 4 helices contained in the α-domain and β-sheets that form a β-domain. However, α-LA has a tightly bound Ca^2+^ in the calcium-binding loop. Removal of Ca^2+^ leads to a molten globule state of α-LA [3], [4]. X-ray crystallographic studies on the complex of α-LA with β4Gal-T1 [5], together with enzyme kinetics studies have led to an understanding of the modulation mechanism in the LS complex [6], [7].

α-LA is expressed only in mammals and in the mammary gland during lactation to function as a lactose synthase complex. However, some breast cancer cells have been found to express α-LA protein [8]-[10]; α-LA has also been shown to cause apoptosis of mouse and human mammary epithelial cell lines [11] as well as of fur seal primary mammary cells, identifying it as a milk factor that regulates involution [12]. Small but detectable amounts of α-LA have been found during the early gestation phase in rat mammary gland [13]. We have also cloned the human α-LA from a cDNA library prepared from the non-lactating mammary gland that lactated previously and have used in the present studies. Thus, these studies indicate that α-LA has been at least transcribed in the breast tissues at various stages, though its function at those stages is not known.

Since 1995, pioneering work by Dr. Svanborg's group has shown that α-LA in the molten globule state complexes with oleic acid (OA), acquiring apoptotic properties toward tumor and immature cells, but not toward differentiated cells [14]–[16]. Extensive studies on the biological property of the complex, named HAMLET by Dr. Svanborg's group, an acronym for human a-lactalbumin made lethal to tumor cells, have shown that it induces mitochondrial depolarization and cytochrome c release [17] and that the apoptotic response triggered by HAMLET is independent of caspase inhibition, p53 status, and Bcl-2 over expression [18]. In addition, HAMLET-induced changes are compatible with macroautophagy [19]. Other studies suggested perturbation of the proteasome structure [20] and lipid membrane integrity [21]. Besides the broad evidence of HAMLET's anti-tumor activity, http://www.ncbi.nlm.nih.gov/pubmed/19048621?ordinalpos=2&itool=EntrezSystem2.PEntrez.Pubmed.Pubmed_ResultsPanel.Pubmed_DefaultReportPanel.Pubmed_RVDocSumthe mechanism(s) of cytotoxicity has not yet been elucidated [22].

After having first cloned α-LA and β4Gal-T1 genes [23], [24] and studied their molecular interactions [5]–[7], here we have initiated the structure-based analysis of HAMLET using our site-specific labeling technique [25], [26]. We have reproduced the previously described results by Svanborg et al., [14]–[16], showing that the molten globule α-LA in complex with OA kills many different tumor cells, but not untransformed or normal cells. We have designed a human α-LA (hLA) and an α-domain of hLA (αD-hLA) with a polypeptide tag at the C-terminal end, hLA-ext and αD-hLA-ext, respectively, which can be specifically glycosylated with ppGalNAc-T2, transferring a modified galactose with a chemical handle (C2-keto-galactose), as described previously [25], [26], that can be further labeled with aminooxy-Alexa Fluor 488. We show here that the tumoricidal complex derived from the site-specific-labeled hLA-ext or αD-hLA-ext also kills many tumor cells and have used these labeled proteins for cell imaging.

## Results

### Protein expression and folding

**Figure 1:**
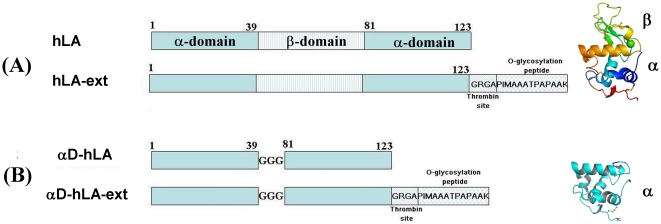
Schematic diagram of the hLA and αD-hLA proteins with C-terminal extension. (A) hLA (protein sequence acc. No J00270) and hLA-ext proteins with the crystal structure of hLA (far right) (pdb 1a4v) showing alpha and beta domains. (B) αD-hLA and αD-hLA-ext proteins with the corresponding alpha domain structure (model) (far right). Since the alpha domain is comprised of N-terminal residues, 1 to 39, and the C-terminal residues, 81 to 124, of hLA (cyan colored), a three residue glycine linker was used to fuse the N-terminal and C-terminal fragments to construct the alpha domain form of the protein (αD-hLA) where beta domain is removed. A 17-amino-acid extension containing a thrombin cleavage site and a Thr residue (substrate for ppGalNAcT2 enzyme), was engineered at the C-terminal domain of hLA (A, hLA-ext) and αD-hLA (B, αD-hLA-ext).

**Figure 2:**
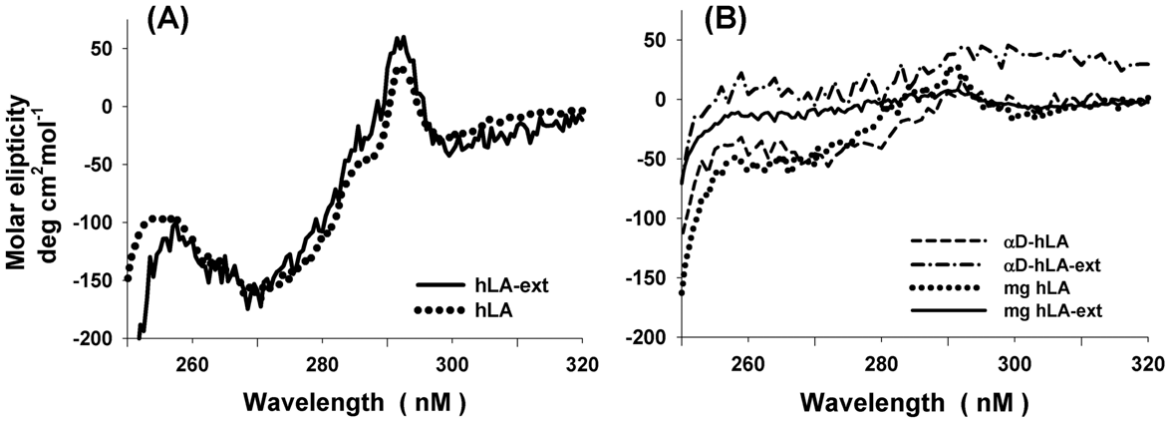
Tertiary structure studied with near UV CD spectra. (A) The near UV spectra of the native refolded hLA and hLA-ext proteins show positive and negative peaks indicating the CD bands arising from aromatic amino acids and suggest the presence of tertiary structure. When these proteins are treated with a chelating agent to remove Ca^2+^ ion, these proteins acquire a molten globule state (B). Interestingly the αD-hLA and αD-hLA-ext show similar near UV CD spectra as molten globule hLA.

**Figure 3:**
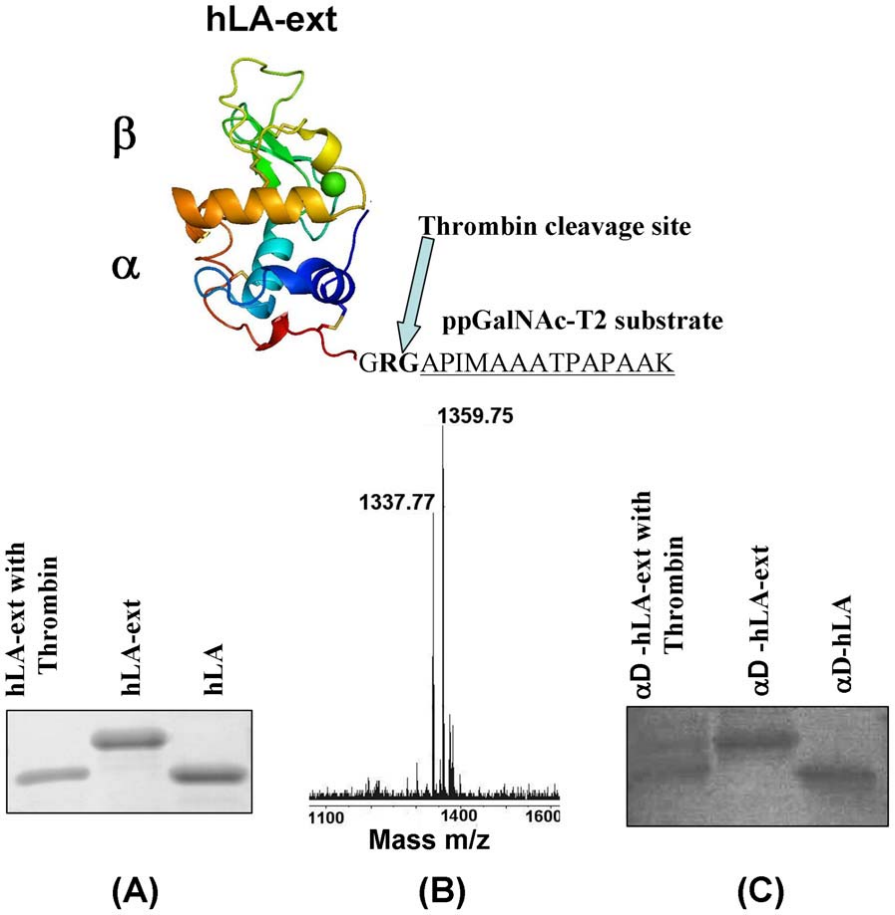
Incorporation of a polypeptide tag to full length hLA and αD-hLA proteins. Schematic representation of the C-terminal extension of 17 amino acids engineered on hLA showing the acceptor Thr residue for the site-specific labeling and the thrombin cleavage site. (A) SDS-PAGE analyses of purified hLA, hLA-ext, with and without thrombin treatment. To confirm the presence of the C-terminal extension peptide, the protein was treated with thrombin. The mobility of hLA-ext treated with thrombin is comparable to wild-type hLA. (B) Since the molecular weight of the released C-terminal polypeptide is only 1337 Da it could not be observed on any SDS-PAGE gel. However, MALDI-TOF spectroscopic analysis of the thrombin treated samples showed a release of a correct molecular weight peptide from the proteins carrying the C-terminal extension peptide. (C) The protein samples αD-hLA and αD-hLA-ext with and without Thrombin treatment were analyzed on SDS-PAGE similar to hLA-ext shown in (A).

The recombinant hLA (124 aa) and hLA with a 17 amino acid C-terminal extension (hLA-ext) (Figure 1A) were expressed in *E. coli* as inclusion bodies and folded *in vitro* as described previously [27], [28]. The near UV CD spectrum show that these proteins exist in their native state with significant tertiary structure (Figure 2A). However when the bound calcium ion is removed by dialysis against EGTA, they adopt a molten globule structure as judged by its near UV spectrum (Figure 2B). The native α-LA contains alpha (α-) and beta (β-) domains (Figure 1A, 3). The alpha-domain (83 aa) comprises of the N-terminal region, residues 1 to 39, and the C-terminal region, residues 81 to 124, of the human LA. We have engineered the α-domain-form of hLA with 86 amino acids (MW 9.7 kDa) (αD-hLA), in which the beta domain has been removed and both fragments of the N- and the C-terminal region were linked by three glycine residues (Figure 1B) [29]. The α-domain hLA protein with and without the C-terminal extension were “refolded” in the absence of calcium salt and purified by ammonium sulfate precipitation. The near UV CD spectrum of these proteins shows that they exist in the molten globule form (Figure 2B).

Although DNA sequencing of the hLA-ext and αD-hLA-ext protein genes confirmed the presence of the C-terminal extension, its presence in these proteins could be clearly seen from the SDS-PAGE gels as these proteins have a higher molecular weight compared to their respective native protein (Figures 3A and C). These gels further showed that upon the loss of the C-terminal extension peptide with thrombin protease treatment their molecular weight is found similar to their respective native protein (Figure 3A and C). Since the molecular weight of the released C-terminal polypeptide is only 1337 Da it could not be observed on any SDS-PAGE gel, therefore, a MALDI-TOF spectroscopic analysis of these thrombin treated samples was carried out. Such analysis shows a release of correct molecular weight peptide upon the treatment of thrombin from these proteins (Figure 3B), confirming the presence of thrombin cleavable C-terminal extension peptide in these proteins.

### Site-specific labeling of hLA-ext and αD-hLA-ext protein molecules

**Figure 4:**
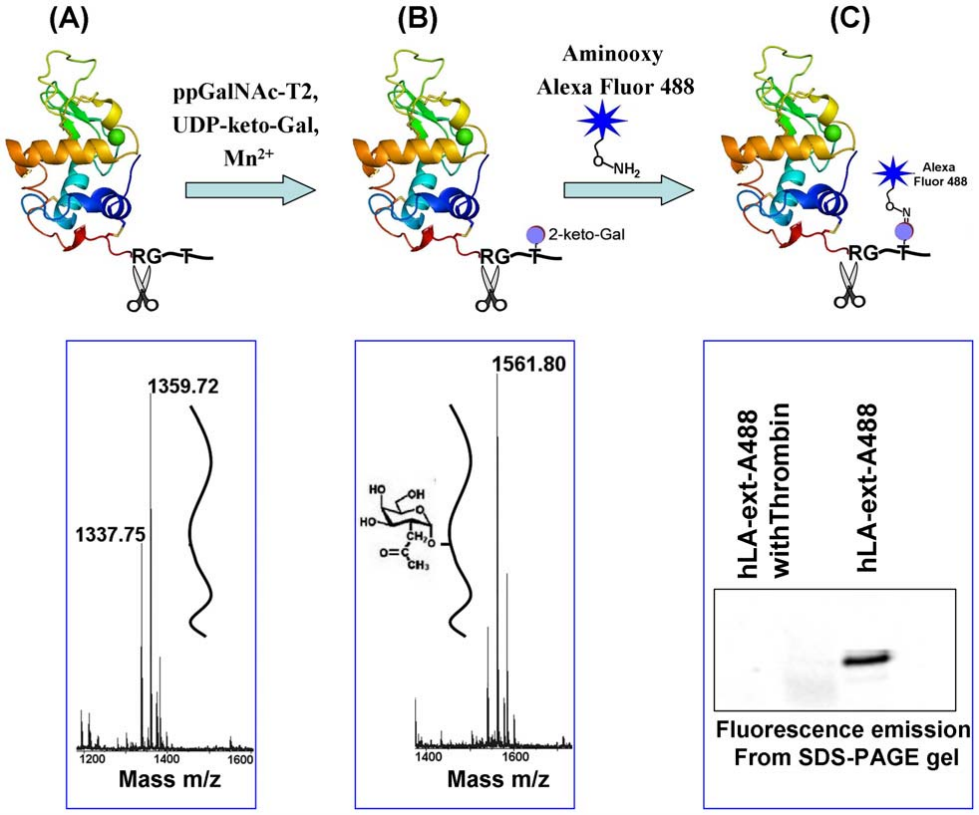
Site-specific labeling with aminooxy-Alexa Fluor 488 of hLA-ext. The hLA-ext protein (A) is glycosylated by the ppGalNAc-T2 enzyme in the presence of Mn^2+^ and UDP-C2-keto-galactose. The lower panel shows the MALDI-TOF analysis of the C-terminal extension peptide released upon thrombin cleavage. (B) The glycosylated hLA-ext protein with a single C2-keto-Gal molecule (blue circle) is conjugated with the aminooxy-Alexa Fluor 488 molecule. The lower panel shows the MALDI-TOF spectrum of the glycosylated C-terminal extension peptide released after thrombin treatment of the glycosylated hLA-ext protein. The increased molecular weight of 201 Da corresponds to a single C2-keto-gal molecule. Since the Alexa Fluor 488 conjugated C-terminal peptide released from the Alexa Fluor 488 conjugated hLA-ext protein could not be observed on a MALDI-TOF spectrum, the protein with and without thrombin treatment was analyzed on a SDS-PAGE gel (C) for the fluorescence emission detection (lower panel). Fluorescence emission from the protein band is only observed when the protein was not treated with the thrombin, suggesting that the Alexa Fluor 488 molecule is conjugated to the C-terminal extension peptide.

Using the ppGalNAc-T2 enzyme, C2-keto-galactose from UDP-C2-keto-Gal was transferred to the single Thr residue, located in the polypeptide extension of the native hLA-ext protein (Figure 4A & B). MS analysis of the thrombin cleaved peptide from the glycosylated hLA-ext protein showed an increase in molecular mass by 201 Da that corresponds to a single C2-keto-gal sugar moiety, thus confirming the presence of a single sugar moiety in the C-terminal extension peptide (Figure 4B). The glycosylated hLA-ext protein was conjugated with aminooxy-Alexa Fluor 488 at pH 3.9 and purified by ammonium sulfate precipitation. Since the thrombin cleaved Alexa Fluor 488 conjugated C-terminal extension peptide could not be analyzed by MALDI-TOF, a fluorescence emission at 488 nM from the Alexa Fluor 488 conjugated hLA-ext protein samples were analyzed on SDS-PAGE gel, with and without thrombin treatment. Only a fluorescence emission from the protein band that was not treated with thrombin is observed, suggesting that the Alexa Fluor 488 molecule is conjugated to the C-terminal extension peptide of the glycosylated hLA-ext protein (Figure 4C). Similarly, the αD-hLA-ext protein is also conjugated with aminooxy-Alexa Fluor 488 molecule after glycosylating with C2-keto-Gal molecule.

**Figure 5:**
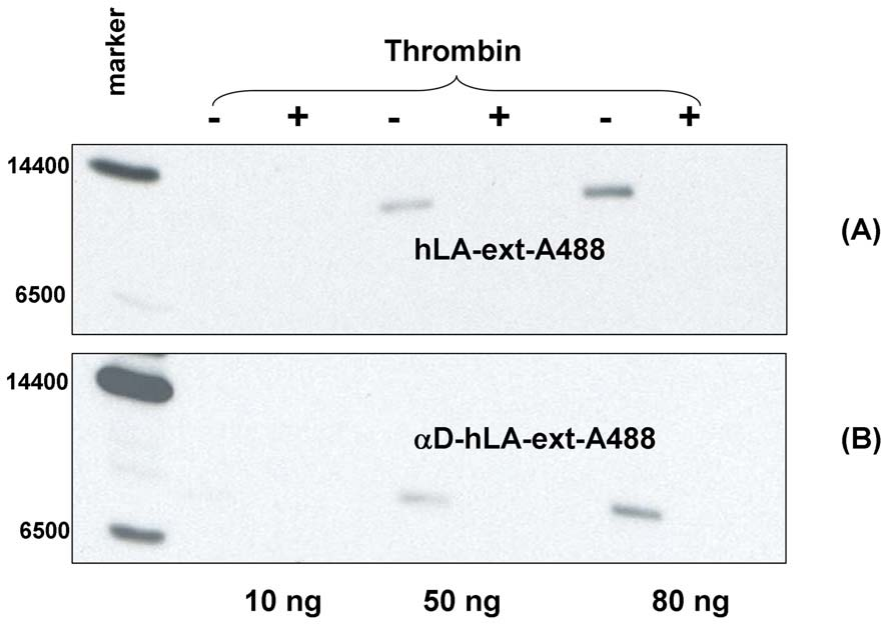
Site-specific labeling of proteins with aminooxy-biotin. Glycosylated proteins of hLA-ext (A) and αD-hLA-ext (B) were coupled with aminooxy-biotin. Proteins were separated on SDS- PAGE and transferred to the nitrocellulose membrane. Biotinylated proteins were detected with streptavidin-HRP and chemiluminescent substrate. Biotinylated HAMLET retained cytotoxic activity (data not shown).

Glycosylated hLA-ext and αD-hLA-ext proteins coupled to aminooxy-biotin molecule were analyzed on non-reduced SDS-PAGE (Figure 5A and B). Various amounts of biotin conjugated protein samples with and without thrombin treatment were run on non-reduced SDS-PAGE gel and transferred to a membrane. The biotin conjugated proteins were detected by streptavidin-HRP and chemiluminescence substrate. Only samples that were not treated with thrombin showed a chemiluminescence band at the expected molecular weight, indicating that these proteins could also be site-specifically biotinylated in the C-terminal extension peptide.

### HAMLET preparation and characterization

HAMLET has been traditionally prepared by passing the apoprotein through an OA-conditioned anion exchange chromatographic column [16]. Recently, Kamijima et al. have used a method for preparing HAMLET that involves mixing and heating the OA with the native protein [30] and confirmed by others [31]. We followed the heating method for the complexation with OA but used the Ca^2+^-depleted hLA (apoprotein) instead of Ca^2+^-bound hLA (holo form) during heating for 10 min at 60^o^C.

**Figure 6:**
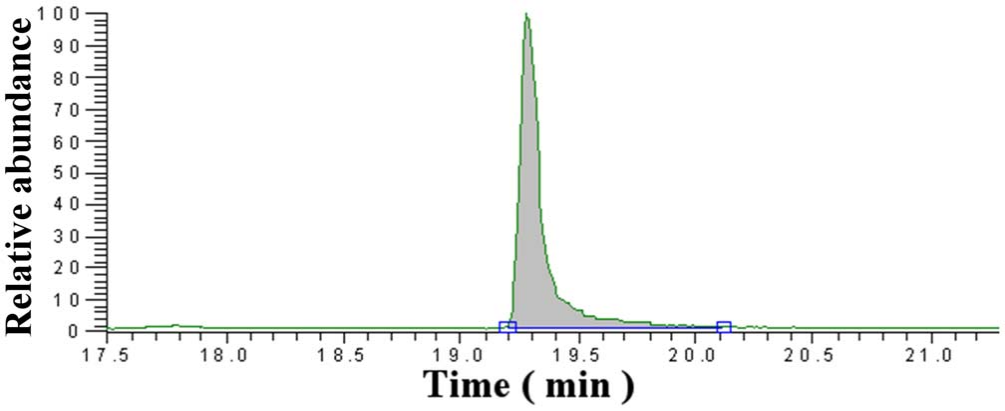
Liquid chromatography–mass spectrometry (LC-MS)/Single Ion Monitoring (SIM) chromatogram. Representative Profile for the Oleic Acid-Protein Complex and its 18 C^13^ isotopic Oleic Acid Internal Standard (ISTD). The original Complex was diluted 60 times with ISTD at 0.5 µg/mL. 25 µL sample solution was injected on to the column.

Protein was estimated by the Bradford method, and fatty acid content was determined using liquid chromatography coupled to a mass spectrometer (Figure 6). Two forms were tested: the molten globule hLA (mg-hLA) and the αD-hLA complexed with OA. The protein to OA molar ratios on various samples were determined by LC-MS studies and listed in Table 1. Previously, Petterson-Kastberg et al. [32] investigated the ratio of protein to OA in HAMLET, and, although the method for preparing HAMLET was the conditioned column, their ratio seems to agree with the results reported here. Using ^1^H NMR spectroscopy, α-LA to OA ratio of 1∶5.4 was observed in HAMLET, and for rhLA^all-Ala^ to OA, ratios were 1∶7.3 (using chemical analysis) and 1∶9.5 (using ^1^H NMR spectroscopy) [32]. In our current study all the protein-OA complexes are made with 1∶10 ratio and the protein to OA ratio determined by the LC-MS method is comparable to the values found by others. Thus the presence of the C-terminal extension with a sugar or sugar conjugated with Alexa fluoro probe does not significantly affect the protein to OA ratio. However, when the complex is made with higher amount of OA (1∶100) the protein-OA complex could not be precipitated with 1M sodium chloride and purified easily. Interestingly when the protein-OA complex is prepared with 1∶25 ratio, the purified complex had a ratio of 1∶35 as determined by the LC-MS. This higher OA value suggests that at higher OA concentrations the protein-OA complex associates with OA and could not be easily purified.

**Table 1:** Oleic acid content determined from the HAMLET preparations using LC-MS method.

Protein	Protein to OA ratio	
	Used to make HAMLET	Determined by LC-MS method
αD-hLA	1∶10	1 ∶10.4
mg-hLA	1∶10	1∶8.2
mg-hLA-ext	1∶10	1∶9.2
mg-hLA-ext	1∶25	1∶34.5
mg-hLA-ext-keto	1∶10	1∶6.1
mg-hLA-ext-keto-Alexa Fluor 488	1∶10	1∶7.1

### Tumoricidal activity by the protein–OA complex

The tumoricidal activity of mg-hLA, mg-hLA–OA complex, mg-hLA-ext, and mg-hLA-ext–OA complex was tested on several tumor cell lines and on immortalized, non-transformed mammary epithelial cell line MCF-10A (Table 2). Detailed studied were carried out with SK-BR-3 and MDA-MB-468 cell lines. Viability was investigated using Trypan blue and Annexin V methods.

**Table 2:** Cell lines tested for the tumoricidal activity with HAMLET prepared with hLA-ext in 1∶10 oleic acid ratio.

Cell lines	Tumoricidal activity HAMLET
SK-BR-3 (breast adenocarcinoma)	+
MDA-MB-468 (breast adenocarcinoma)	+
Jurkat (T-cell lymphoma)	+
A549 (lung adenocarcinoma)	+
MCF-7 (breast adenocarcinoma)	+
U-87 (glioblastoma)	+
HeLa (cervical adenocarcinoma)	+
MCF-10A (immortalized, non-transformed mammary epithelial cells)	-

**Figure 7:**
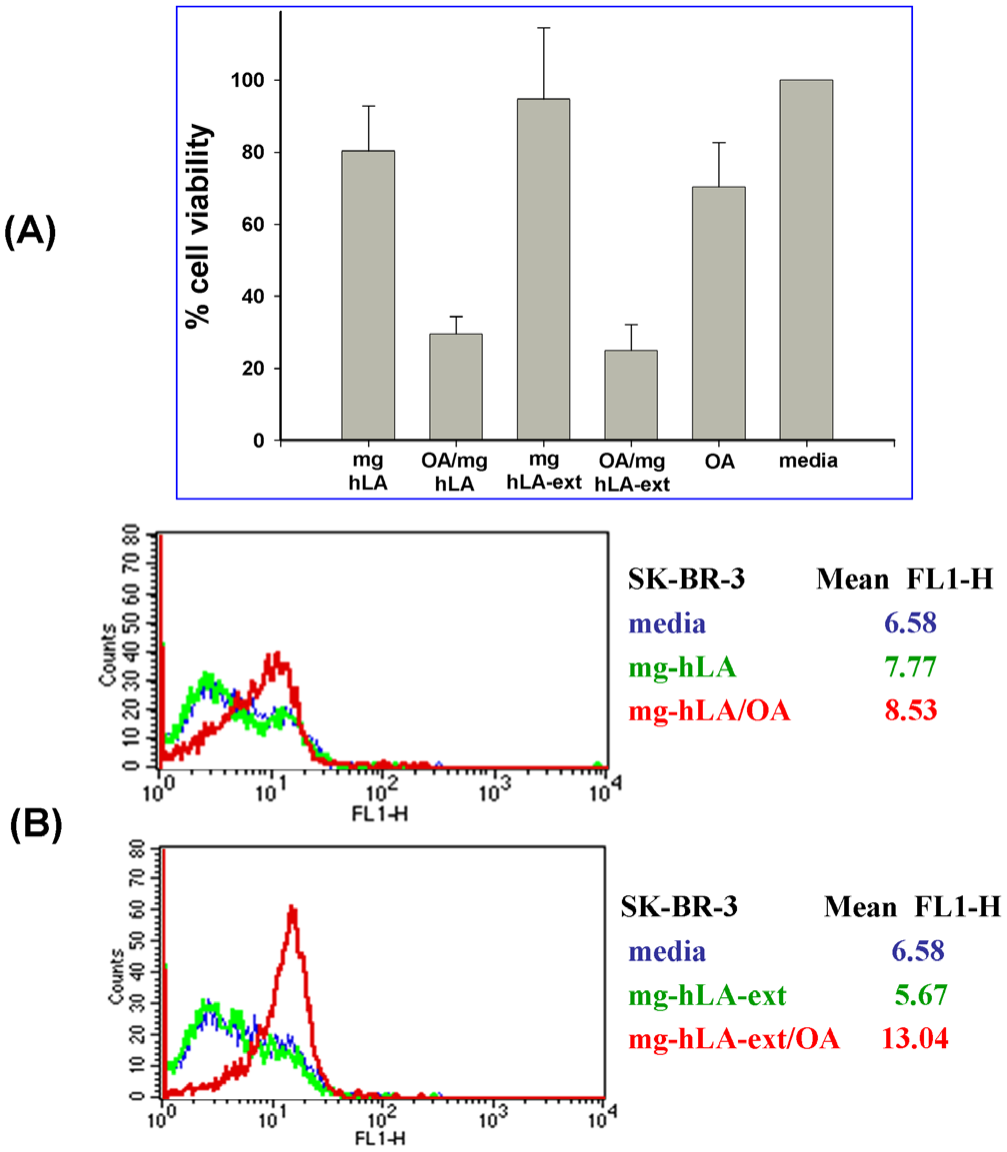
Measurement of tumoricidal activity during conversion of recombinant protein to tumoricidal complex. The effect on the cell viability of SK-BR-3 cells was studied after treatment with mg-hLA, mg-hLA/OA, and mg-hLA-ext and mg-hLA-ext/OA. The cells were incubated with molten globule proteins alone or complexed with OA, and their viability was investigated using Trypan blue (A) and by FACS analysis with Annexin V (B). When cells were incubated with the apo-form of hLA (mg-hLA and mg-hLA-ext), viability was comparable to the control (media alone) in the cell lines studied. When molten globule protein derived from hLA or hLA-ext was complexed with OA in a 1∶10 ratio, viability of the cell lines SK-BR-3 was reduced. Results are shown as mean ± SEM (*p<0.05).

SK-BR-3 cells (Figure 7) and MDA-MB-468 cells (data not shown) were incubated for 3 h with 30 µM molten globule proteins (mg-hLA or mg-hLA-ext) alone or in complex with OA (1∶10 ratio). Control cells were incubated with media only, and their viability was considered to be as 100%. Control cells were incubated with 300 µM OA alone. Three independent experiments were performed, with results shown as mean ± SEM (*p<0.05) (Figure 7). When cells were incubated with the mg-hLA, viability in the cell lines studied was comparable to the control (media alone). The same results were obtained with the molten globule protein of the modified hLA with a C-terminal extension (mg-hLA-ext). When mg-hLA was complexed with OA in a 1∶10 ratio, viability of the SK-BR-3 cell lines was reduced by 70% (Figure 7A). The same effect was observed with the complexes derived from the mg-hLA-ext, indicating that the C-terminal portion of the hLA does not interfere with its tumoricidal activity (Figure 7A). Incubation with OA alone had a mild influence on the viability of the SK-BR-3 cells. Similar results were obtained with the MDA-MB-468 cells (data not shown). The tumoricidal activity was studied using Annexin V-FITC staining of the cell lines SK-BR-3 (Figure 7B) and MDA-MB-468 (data not shown) after exposure to hLA in a molten globule state complexed with OA. Cells were incubated for 1 h with 30 µM protein and then stained according to the manufacturer's protocol. Five-thousand events were analyzed by flow cytometry. Phosphatidylserine exposure (detected cell surface by Annexin V binding) was higher in cells incubated with OA complexes derived from mg-hLA and mg-hLA-ext than that in cells incubated with protein without complex with OA or media alone, as evidenced by the shift in fluorescence emission (Anexin V-FITC). In conclusion, the OA complexes of both mg-hLA and mg-hLA-ext-showed comparable tumoricidal activities. The addition of a 17–amino acid C-terminal tag to hLA doesn't affect its biological activity when complexed with OA, making it a suitable substrate for subsequent labeling.

In addition, we have tested other human cell lines, Jurkat (T-cell lymphoma), A549 (lung adenocarcinoma), MCF-7 (breast adenocarcinoma), U-87 (glioblastoma), and HeLa (cervical adenocarcinoma) (data not shown), and studied the specificity of the complex toward tumor cell lines and the MCF-10A immortalized, non-transformed mammary epithelial cells. We found the complex is not cytotoxic when MCF-10A cells were cultured to confluence (data not shown), as previously described for other non-tumor cells [14].

### Confocal microscopy studies

**Figure 8:**
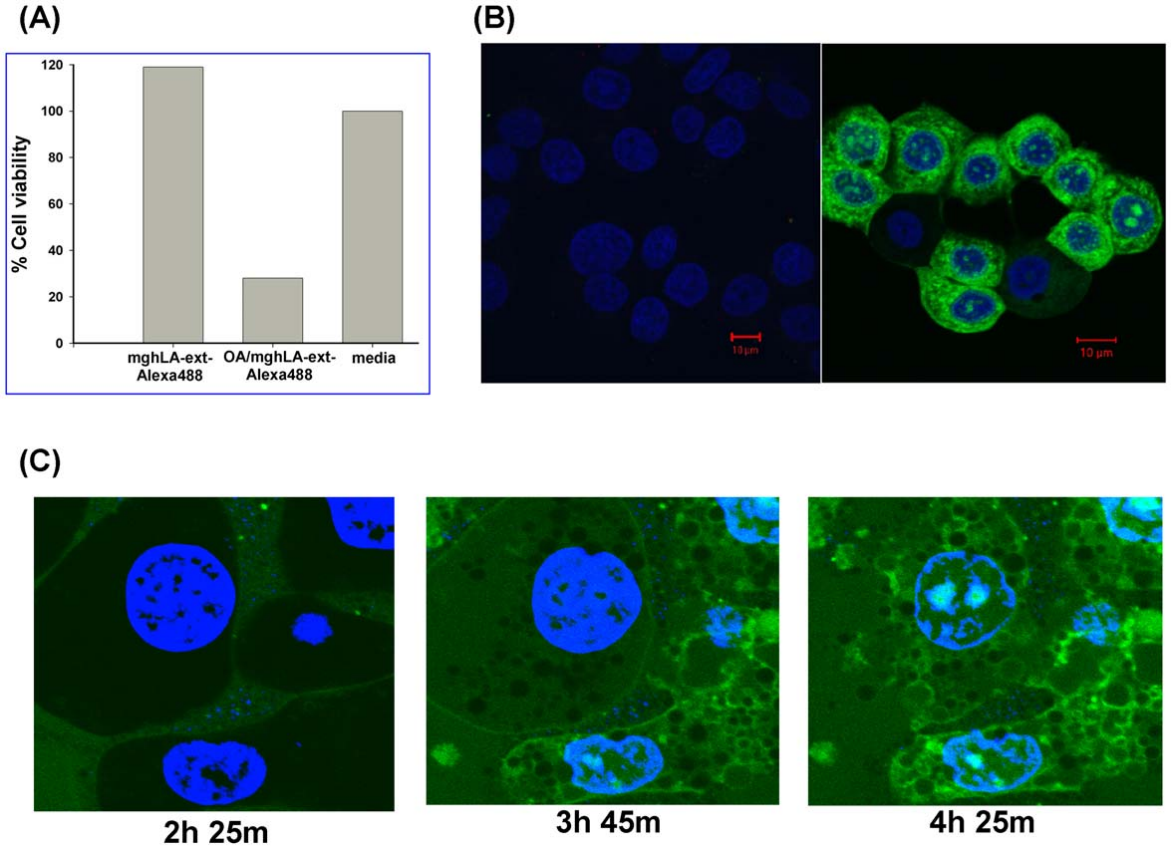
Microscopy studies using site-specific-fluoroprobe-labeled protein. (A) Tumoricidal activity of the labeled protein hLA-ext in molten globule state was tested on A549 cells. MCF-7 cells were incubated for 4 h, with 30 µM Alexa Fluor 488-labeled mg-hLA-ext protein alone (B) or complexed with OA (C). After treatment, cells were fixed and nuclei were stained with vital stain Hoechst 33342 (blue). When complexed with OA, Alexa Fluor 488 labeled mg-hLA-ext (green) internalizes to cytoplasm and cell nuclei, while molten globule protein alone did not enter the cells. (D) Confocal images of SK-BR-3 cells treated with Alexa Fluor 4888-labeled mghLA-ext complexed with OA at the indicated times.

For confocal microscopy studies, we labeled the C-terminal extension of hLA-ext first with C2-keto-Gal and then conjugated it with Alexa Fluor 488 (Figure 4). The labeled complex tested on A549 cells showed tumoricidal activity (Figure 8A).Using the labeled complex or molten globule protein alone; we studied internalization of the mg-hLA-ext by fluorescence confocal microscopy on an MCF-7 cell line (Figure 8). We found that the Alexa Fluor 488–labeled mg-hLA-ext when complexed with OA (Figure 8C) internalizes to cytoplasm and cell nuclei, while the Alexa Fluor 488–labeled molten globule protein of hLA-ext alone was not internalized (Figure 8B).

The internalization process could be followed by live confocal microscopy on SK-BR-3 cells (Figure 8D). SK-BR-3 cells were treated with mg-hLA-ext-Alexa Fluor 488/OA (green). Nuclei were stained in blue with Hoechst 33342. With time, the complex bound to the cell membrane and was internalized. Accumulation of a green fluorescent signal could be detected in the cytoplasm and cell nuclei. The DNA was fragmented when this process occurred, and chromatin was seen on the nuclear margins. Inside the cell nucleus, an accumulation of green fluorescent signal at defined structures was seen.

**Figure 9:**
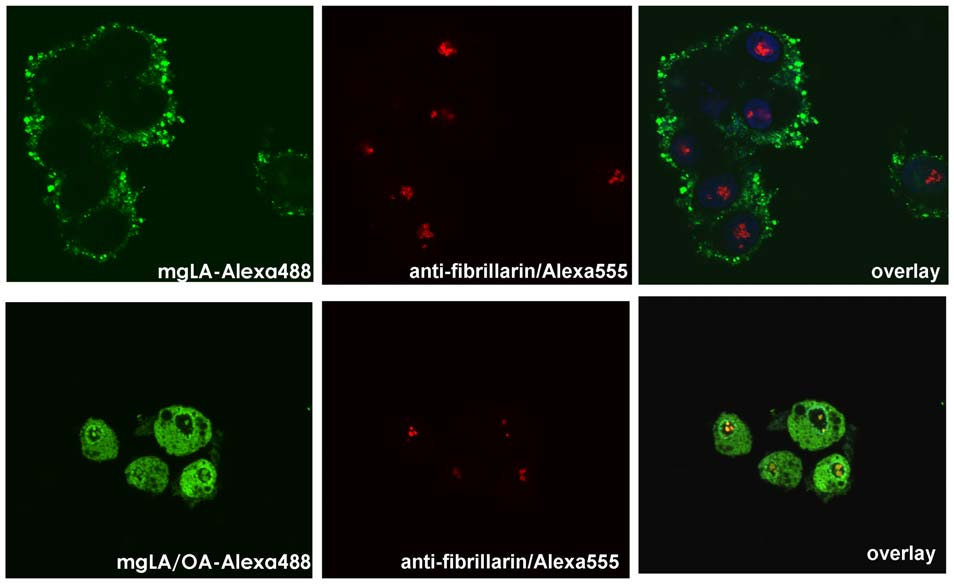
Aggregates in the nuclei co-localize with a nucleolus marker. SK-BR-3 cells were treated with Alexa Fluor 488 labeled mg-hLA-ext complexed with OA or without OA (control) for 4 h, fixed, permeabilized, incubated with a-fibrillarin monoclonal antibody, and detected with anti-mouse-Alexa Fluor 555–labeled antibody (red). In cells treated with OA complex, co-localization of green fluorescent signal and fibrillarin protein was found at the cell nuclei in SK-BR-3 and MDA-MD-468 (data not shown) cell lines.

In a different study, co-localization of the green fluorescent signal corresponding to the complex and fibrillarin protein was found at the cell nuclei in SK-BR-3 cells (Figure 9) and MDA-MB-468 cells (data not shown) treated with mg-hLA/OA complex.

### α-Domain hLA (αD-hLA)

**Figure 10:**
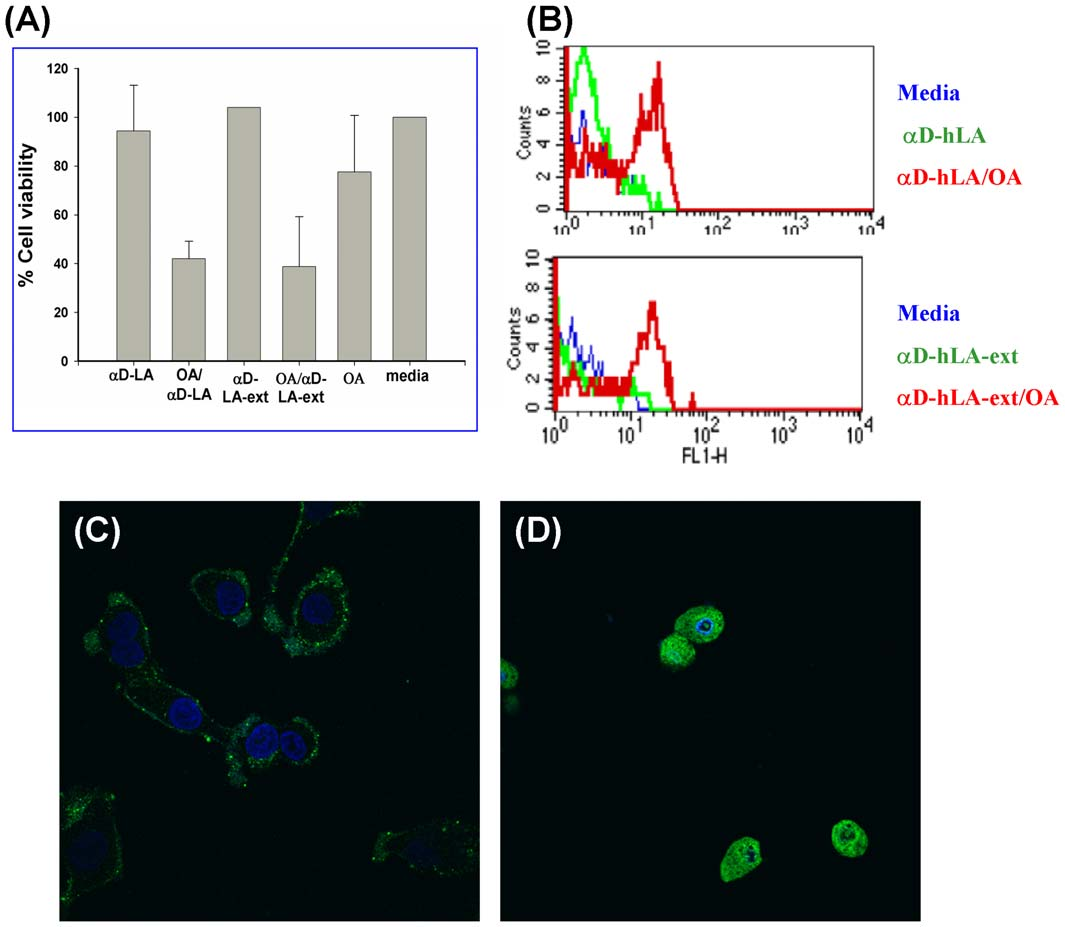
Reduced size HAMLET has comparable properties to complex derived from full-length protein. αD-hLA in complex with OA has biological properties that are similar to mg-hLA OA complex with cancer cell lines SK-BR-3 and MDA-MD-468 (data not shown). αD-hLA and αD-hLA-ext, complexed with OA have comparable tumoricidal activities measured by Trypan Blue (A) and by FACS analysis using Annexin V (B), indicating that the β-domain of hLA is not required for the complex to acquire cytotoxic properties. SK-BR-3 cells were incubated with Alexa Fluor 488 labeled αD-hLA-ext either alone (C) or as OA complex (D) for 3 h. The cells were then fixed with 4% PFA in PBS, and the nuclei were counterstained with Hoechst 33342 (blue). Only αD-hLA-ext-Alexa Fluor 488 (green signal) complexed with OA is internalized by the cells.

Alpha domain of hLA (αD-hLA) with and without OA complex were tested on SK-BR-3 cells and MDA-MD-468 cells (data not shown) and biological activities were observed using Trypan Blue and Annexin V (Figure 10 A and B). The αD-hLA and αD-hLA with extension (αD-hLA-ext) complexed with OA showed comparable tumoricidal activities, indicating that the beta domain of hLA is not required for the complex to acquire cytotoxic properties. The αD-hLA-ext was glycosylated, labeled with Alexa Fluor 488, and complexed with OA. To visualize the internalization of this alpha domain–derived complex, cells were incubated with labeled proteins, with (Figure 10D) and without (Figure 10C) complexation with OA and fixed. Nuclei were stained with Hoechst 33342. Only complexed protein was internalized after 3 h of incubation. These results indicate that we have been successful in expressing, labeling, and imaging, by confocal microscopy, a reduced size HAMLET-like molecule.

## Discussion

Since the discovery 16 years ago of HAMLET [14], a tumoricidal complex originally isolated from human milk, investigators have been challenged to deepen the knowledge of its nature. Extensive work has already been achieved, such as defining the need for both components, the molten globule state of α-LA and OA as a lipid cofactor, and defining the cellular targets, which include mitochondria [17], proteasomes [20], nuclei [33], [34], lysosomes [35], and the cell membrane [21]. Still, many aspects remain unexplained, such as HAMLET's specificity for tumor cells, the possible presence of a HAMLET receptor, possible mechanism of action, and the nature of the complex's structure.

Studies have shown that α-LA without any disulfide bond, where all the cysteine residues are mutated to alanine residues, or various protease digested fragments of native α-LA can also form a tumoricidal complex with OA [32], [36]. Earlier protease digestion and spectroscopic studies on the molten globule state of α-LA indicated that the β-domain of the protein may be more disordered than the α-domain [37]. Furthermore, the deletion of the β-domain from α-LA has been shown to have little impact on the tertiary structure of the α-domain [29]. Therefore, we have chosen the α-domain of the hLA protein for further investigation in the present study. Like the full-length protein, the α-domain, after refolding also remained a monomer and formed a tumoricidal complex with OA.

In all studies, the molten globule state of the α-LA was found to be essential for forming a tumoricidal complex with OA. It has been shown that, in the molten globule state α-LA is more hydrophobic compared to its holo-form [38]. Thus, this state may facilitate the binding of the fatty acids, such as OA. A simple addition of OA to the molten globule α-LA solution may not be enough to make the complex, as most OA molecules exist as micelles in water. Simple heating of such a solution may disrupt the micelles enough to cause a few OA molecules to dissociate and bind to a molten globule α-LA molecule, thus generating a HAMLET molecule. Although, it may be hard to predict how many OA molecules may be bound to the hydrophobic surface of a α-LA molecule, the number we have determined in our complex is comparable to the ones reported by others.

We have earlier developed a site-specific labeling technique of proteins. In this technique, the target protein is made with a C-terminal 17–amino acid fusion peptide. This peptide contains a single Thr residue to which a C2-keto-galactose sugar is transferred from its UDP-derivative by the ppGalNAc-T2 enzyme [26]. Since the glycosylated protein carries a unique chemical handle at its sugar moiety, it can be used for site-specific conjugation of a bioactive agent with an aminooxy group. In the present study, extending the α-LA with C-terminal fusion peptide was not expected to alter its biological activity, since α-LA from rat naturally exists with an 11–amino acid C-terminal extension [23]. The labeling was carried out before the OA complex was formed. We have observed that the native hLA with the N-terminal his-tag readily binds to a metal affinity column; however, after the OA complex is made, the protein does not bind to the metal affinity column. Therefore, the C-terminal labeling was carried out prior to making the OA complex. Furthermore, the C-terminal extension of the native α-LA by itself does not affect its activity either as lactose synthase (data not shown) or, as shown in the present study, as a HAMLET, indicating that the protein with the C-terminal extension carrying the bulky fluoroprobe can still be converted into tumoricidal complex.

Fluoroprobe-labeled and complexed molten globule protein was used for live-/fixed-cell imaging. Alexa Fluor 488 signal could be detected at the cell surface and, with time, accumulating at the cell nucleus, as shown by Hakansson et al. [33]. Moreover, we have identified a subnuclear structure in which the green fluorescent signal of Alexa Fluor 488 accumulated in nucleoli, using anti-fibrillarin antibody. The significance of co-localization of a labeled complex with cell nucleoli is unknown. However, a variety of molecules that apparently have no role in ribosome assembly, have been found at nucleoli [39]. In addition, one study has suggested that unfolded proteins are stored in the nucleolus during stress [40].

In addition to using site-specific labeling for microscopy studies, we have shown that the site-specific biotin coupling could also be achieved, suggesting a potential application for ultra-structural studies, such as transmission electron microscopy. Biotinylated protein could be used to “fish-out” cancer cell surface ligands interacting with HAMLET. These specific interactions between the cell surface ligand(s) and HAMLET may be enough to initiate the apoptotic process(es) leading to tumor cell death.

In conclusion, we have been successful in adding a chemical handle to HAMLET without affecting its biological activity, allowing us to trace the complex at the cell surface or inside the cell. The addition of a site-specific handle to a tumoricidal complex could be further exploited for isolation of partner/target molecules, providing insight into the tumor specificity of the complex.

## Materials and Methods

### Cloning, expression, and refolding of hLA, hLA-ext, T-hLA, T-hLA-ext, and His-hLA

The human α-LA gene was cloned from a mammary gland cDNA library (Clontech, Mountain View, CA) into *Nde* I and *Bam*H I restriction sites of the modified pET17 expression vector, pETnef, similar to mouse α-LA as described earlier [27]. The hLA-ext and hLA with N-terminal Histidine-tag (His-hLA) were constructed in *Nde* I and *Eco*R I restriction sites of the pETnef vector, similar to the glutathione S-transferase protein with the same C-terminal extension, as described previously [26]; the deletion of the β-domain in the human α-LA, the αD-hLA, was constructed as described previously [28]; the construction of αD-hLA with C-terminal extension (αD-hLA-ext) was similar to the previously published method [26]. From 1 L of bacterial culture, 15 mg of αD-hLA, and 5.32 mg of αD-hLA-ext were obtained.

All the clones were sequenced and transfected into BL21 DE3LysS cells for protein expression. The expression and refolding of these proteins were carried out under conditions similar to those described previously for mouse α-LA [27], [28]. Nearly 5 mgs and 6 mgs, of purified protein were obtained from 1 L of bacterial culture for hLA and hLA-ext, respectively. For His-hLA purification, after folding protein was dialyzed first against 10 mM Tris-HCl (pH 8.0) and then against PBS, and next purified using TALON metal affinity resin (Clontech). Protein was eluted from the column in PBS buffer containing 1 M NaCl and 100 mM imidazol (pH 7.4). Elution buffer was removed by dialysis against 20 mM Tris-HCl (pH 8.0).

### Confirmation of the C-terminal extension with thrombin cleavage

Seventeen micrograms of purified hLA-ext was incubated overnight at room temperature with one unit of thrombin from human plasma from Sigma-Aldrich (St Louis, MO) in a thrombin reaction buffer (10 mM Tris-HCl (pH 8.0), 2 mM CaCl_2_, and 150 mM NaCl). Equal amounts of cut and uncut proteins were analyzed by 18% SDS-PAGE and by mass spectrometry.

### Circular dichroism spectroscopy

Near (320 to 250 nm) UV circular dichroism spectroscopy (CD) spectra were measured with AVIV Mod.202 CD Spectrometer Aviv Instruments (Lakewood, NJ) with a 10-mm light path CD UV Hellma quartz cell (Plainview, NY). The protein concentration was measured by absorbance at 280 nm. All measurements were made at 25^o^C. The wavelength step was 1 nm, with response time 5 sec; one scan was performed, and the waiting time was 0.5 sec. The spectrum of the pure buffer was subtracted from the protein spectra. The molar ellipticity T (mdeg x cm^2^ x dmol^−1^) was calculated from milidegree, the number of amino acids (aa), protein concentration (c, in molar units), and cell length (l, in cm) as T  =  milidegrees/ (aa x c x l).

### α-Lactalbumin conjugation and labeling

Recombinant ppGalNAcT2 enzyme was prepared as previously described [5].Forty µg of hLA-ext was glycosylated with 20 µg of ppGalNAc-T2 overnight at room temperature in the presence of 25 mM Tris-HCl (pH 8.0), 10 mM MnCl_2_, and 0.5 mM UDP-C2-keto-Gal in a total volume of 100 µL. To label hLA-ext with the fluorescent probe, 7 µL of 10 mg/ml C2-keto-Gal glycosylated hLA-ext protein were labeled in a 40-µL volume containing 166 mM sodium acetate, pH 4.9, and 0.675 mg/ml aminooxy-Alexa Fluor 488 (Invitrogen, Carlsbad, CA). The reaction mixture was incubated overnight in the dark, at room temperature, in a rocking platform. Excess Alexa Fluor 488 was removed by ammonium sulfate precipitation of the labeled protein. Excess ammonium sulfate was removed by washing with 20 mM Tris (pH 8) in 10,000 MWCO centrifugal filters (Amicon, Co. Cork, Ireland).To verify that labeling occurred in the extension sequence of hLA-ext, a 200 fold dilution of the labeled hLA-ext was incubated with thrombin for 48 h. Cut and uncut protein samples were electrophoresed on 14% SDS-PAGE gel. A fluorescence signal was detected in a Hitachi FMBIOII Multi-View scanner with a 505-nm filter.

To label hLA-ext and αD-hLA-ext with biotin, 2.3 mg of C2-keto-Gal glycosylated hLA-ext and αD -hLA-ext proteins were labeled in a 300-µl volume containing 166 mM sodium acetate, pH 4.9, and 3 mM aminooxy-biotin (Dojindo Laboratories, Japan). The reaction mixture was incubated overnight at room temperature. Excess biotin was removed by washing with 20 mM Tris-HCl (pH 8.0) in Amicon Ultra centrifugal filters with 10,000 MWCO for hLA-ext, and 3,000 MWCO for αD-hLA-ext (Millipore, MA).

To detect the labeled protein, 10-, 50-, and 80-ng samples were separated on an SDS-PAGE. Protein was transferred to a nitrocellulose membrane (Invitrogen), blocked with 5% nonfat dry milk, 0.2% Tween 20 for 1 h at room temperature, and incubated for 1 h with 1∶4000 diluted streptavidin conjugated with horseradish peroxidase (GE Healthcare, Piscataway, NJ) in a PBS solution containing 3% BSA and 0.02% Tween 20. After four 5-min washes with blocking solution, the membrane was incubated with HRP substrate ECL Western Blotting Analysis System (GE Healthcare) and exposed to Kodak BioMax light film. To detect the labeling in the extension tag, samples were treated with thrombin. Control samples remained untreated. Labeled hLA-ext was converted to molten globule and complexed with OA as described below.

### Preparation of hLA–OA, unlabeled and labeled hLA-ext–OA complexes

α-LA–OA complexes were prepared by the method described by Kamijima et al. [30], with following modification: briefly, a 210-µM solution of recombinant hLA was incubated overnight with 5 mM EGTA and dialyzed against 5 mM EGTA/20 mM Tris-HCl (pH 8.0) at room temperature to remove calcium. EGTA was removed by a second dialysis overnight at room temperature against 20 mM Tris-HCl (pH 8.0).

OA (Fluka, Buchs, Switzerland) was initially dissolved in 20% ethanol/80% 20 mM Tris-HCl (pH 8.0) solution and further diluted in 20 mM Tris-HCl (pH 8.0) buffer to give a 24-mM stock solution of OA. A solution of the molten globule protein (210 µM) in 20 mM Tris-HCl (pH 8.0) was mixed with OA stock solution (in a 1∶10 molar ratio of protein to OA), heated for 10 min at 60^o^C, and cooled to room temperature. Excess OA was removed by mild centrifugation. Further, protein (complexed or molten globule alone) was precipitated with NaCl (final concentration 1M) and adjusted to a final pH of 3.5 with 1 M HCl. After 4 h on ice or overnight at 4^o^C, the precipitated complex was re-dissolved in 20 mM Tris-HCl (pH 8.0) and dialyzed overnight against 20 mM Tris-HCl (pH 8.0) to remove excess NaCl. For His-hLA samples, after removal of calcium, complexation with OA was performed as described above.

### Determination of protein to OA ratio

Protein concentration of the complex was determined by the Bradford method, and OA concentration was determined by HPLC-ESI-MS/MS technique using a HPLC (Agilent 1100 series) coupled to triple quadrupole mass spectrometer (TSQ Quantum Discovery Max from Thermo Scientific) utilizing the single-ion monitoring technique in negative ionization mode [41]. For quantitation of OA, stable isotopic OA containing 18 [^13^C]-labeled carbon atoms was used as the internal standard, and was purchased from Cambridge Isotope Laboratories (Andover, MA). OA was monitored at m/z 281.2, and the internal standard (ISTD) was monitored at m/z 299.3. Calibration solution of neat OA was made in 1/1 v/v water:MeOH at 0.01, 0.1, 1, 5, 10, 50, and 100 µg/ml levels containing 0.5 µg/ml internal standard. At the 100 µg/ml calibration level, the peak saturation occurs. 16.67 µl of original protein-OA solution was diluted 60 times to give 1 ml of sample solution that also contained 0.5 µg/ml of ISTD. For quantitative analysis, 25 µl of calibrator or protein-OA sample solution was injected into a LC-MS system comprised of a capillary LC column (Supelco C18 column (50 mm×0.5 mm×5 µm), Sigma) coupled via electrospray to a triple quadrupole mass spectrometer utilizing single ion monitoring technique (SIM) in negative ionization mode, monitoring OA at m/z 281.2, and the internal standard (ISTD) at m/z 299.3. The protein-OA sample was run in triplicate. A linear calibration curve with a weighting index of 1/X was used, and an R^2^ of 0.9999 was observed.

### Cell culture

Human lung adenocarcinoma cells A549 were cultured in RPMI-1640 medium (HyClone, Thermo Fisher, Logan, UT) supplemented with 2 mM glutamine, 100 IU/ml penicillin- 100 µg /ml streptomycin (HyClone), 50 µg/ml gentamicin (Gibco/Invitrogen, Grand Island, NY), 1% non-essential amino acids (HyClone), and 10% (v/v) fetal bovine serum (FBS, Gibco). SK-BR-3 and MDA-MB468 cells were cultured in McCoy's 5A media (Gibco) supplemented with 10% FBS and antibiotics. MCF-7 cells were grown in RPMI 1640 (Gibco) and supplemented with 10% FBS and antibiotics.

MCF-10A immortalized, non-transformed mammary epithelial cells (obtained from Dr. Esta Sterneck's laboratory, NCI-Frederick) were cultured in DMEM/F-12 1∶1 (Gibco) supplemented with 100 IU/ml penicillin- 100 µg/ml streptomycin (HyClone), FBS, 10 µg/ml insulin (Sigma), 100 ng/ml Cholera toxin (Calbiochem, La Jolla, CA), 0.5 µg/ml Hydrocortisone (Sigma), 20 ng/ml EGF (Invitrogen) and 1 mM CaCl_2_.

### Cell death assay

Cell viability was determined using the Trypan blue exclusion method. Cells, 5x10^4^ cells per well, were plated in a 48-well plate (Corning Incorporated, Corning NY). After 24 h, media was removed and replaced with fresh media without FBS, and the different protein/protein complexes were added and incubated for the indicated lengths of time. Cells were incubated with 30 µM molten globule protein alone or complexed with OA for 3 h (the first hour of incubation was without FBS). After treatment, floating cells from conditioned media and trypsinized cells were pooled and pelleted. Cells were resuspended in 50 µL of PBS, and the cell suspension was mixed with an equal volume of 0.4% Trypan blue stain (Gibco). Clear, viable cells were counted microscopically in Kova glasstic slides with grid chamber (Hycor, Garden Grove, CA). Cell number is expressed as a percentage of the untreated control cells.

For Annexin V detection, cells were incubated with 30 µM molten globule protein alone or complexed with OA. After treatment, floating and trypsinized cells were pelleted and washed once with binding buffer and stained with Annexin V-FITC (ApoAlert, Clontech, Mountain View, CA) according to the manufacturer's instructions. Cells were quantified in a Becton Dickinson FACScalibur cytometer and CellQuest software.

### Confocal fluorescence microscopy

Cells were plated on an 18-well slide (ibiTreat, Ibidi Integrated BioDiagnostics, Munich, Germany) and grown for 2 nights (2000 cells/well). Media was replaced with mg-hLA complexed with OA (20 µM unlabelled or 10 µM labeled); or mg-hLA alone (20 µM unlabelled or 10 µM labeled) and cells were then incubated at 37°C in media without serum. Same procedure was followed for αD-hLA.

For anti-fibrillarin staining, cells were incubated for 4 h, and then slides were fixed for 15 min with 4% paraformaldehyde in PBS. After rinsing with PBS, cells were permeabilized in a PBS solution containing 0.1% TritonX-100, 2 mg/ml BSA, and 1 mM sodium azide for 5 min, and then blocked for 1 h in a PBS blocking solution (0.05% Tween 20, 2 mg/ml BSA, 1 mM sodium azide). Samples were incubated overnight with anti-fibrillarin mouse monoclonal antibody (Invitrogen), 1∶500, in blocking solution at room temperature in a humidified chamber. After 3×10-min wash, the sample was incubated with 1∶1000 anti-mouse-Alexa Fluor 555 antibody (Invitrogen) in blocking solution. Following the washes, nuclei were stained with Hoechst 5 µg/ml for 15 min, and slides were mounted with mounting media (Ibidi).

Confocal microscopy studies were performed with an Olympus Fluoview 1000 inverted microscope with lX81. Olympus FV100 2.0c and FV-ASW 1.6 viewer software were used for acquisition and analysis, respectively.
